# Supplementary material for: Prolonging the delivery of influenza virus vaccine improves the quantity and quality of the induced immune responses in mice
Source: Front Immunol. 2023 Oct 5;14:1249902. doi: 10.3389/fimmu.2023.1249902 (PMC10585035; doi:10.3389/fimmu.2023.1249902)
Supplement: Supplementary file 1 [file DataSheet_1.docx]

Supplementary Tables

**Supplementary Table 1**. Antibody Panel

| **Marker** | **Clone** | **Fluorchrome** | **Dilution** | **Company (catalogue number)** |
| --- | --- | --- | --- | --- |
| CD19 | 6D5 | Alexa Fluor 488 | 400x | Biolegend (115521) |
| GL7 | GL7 | Alexa Fluor 647 | 400x | Biolegend (144606) |
| CXCR4 | 247506 | PE | 200x | R&D Systems (FAB21651P-100) |
| CD86 | GL-1 | Brillian violet 421 | 200x | Biolegend (105032) |
| CD4 | GK1.5 | Brillian Violet 786 | 200x | BD Biosciences (563331) |
| CD185 (CXCR5) | SPRCL5 | SuperBright 600 | 100x | Thermo Fisher Scientific (63-7185-82) |
| PD1 | J43 | BUV737 | 200x | BD Biosciences (749422) |
| CD3 | 17A2 | Brillian Violet 510 | 200x | Biolegend (100234) |
| IgD | 11-26c.2a | BUV395 | 200x | BD Biosciences (564274) |
| CD138 | 281-2 | APC-R700 | 200x | BD Biosciences (565176) |
| Viability Dye |  | Zombie NIR | 1000x | Biolegend (423108) |
|  |  |  |  |  |

Supplementary Figures


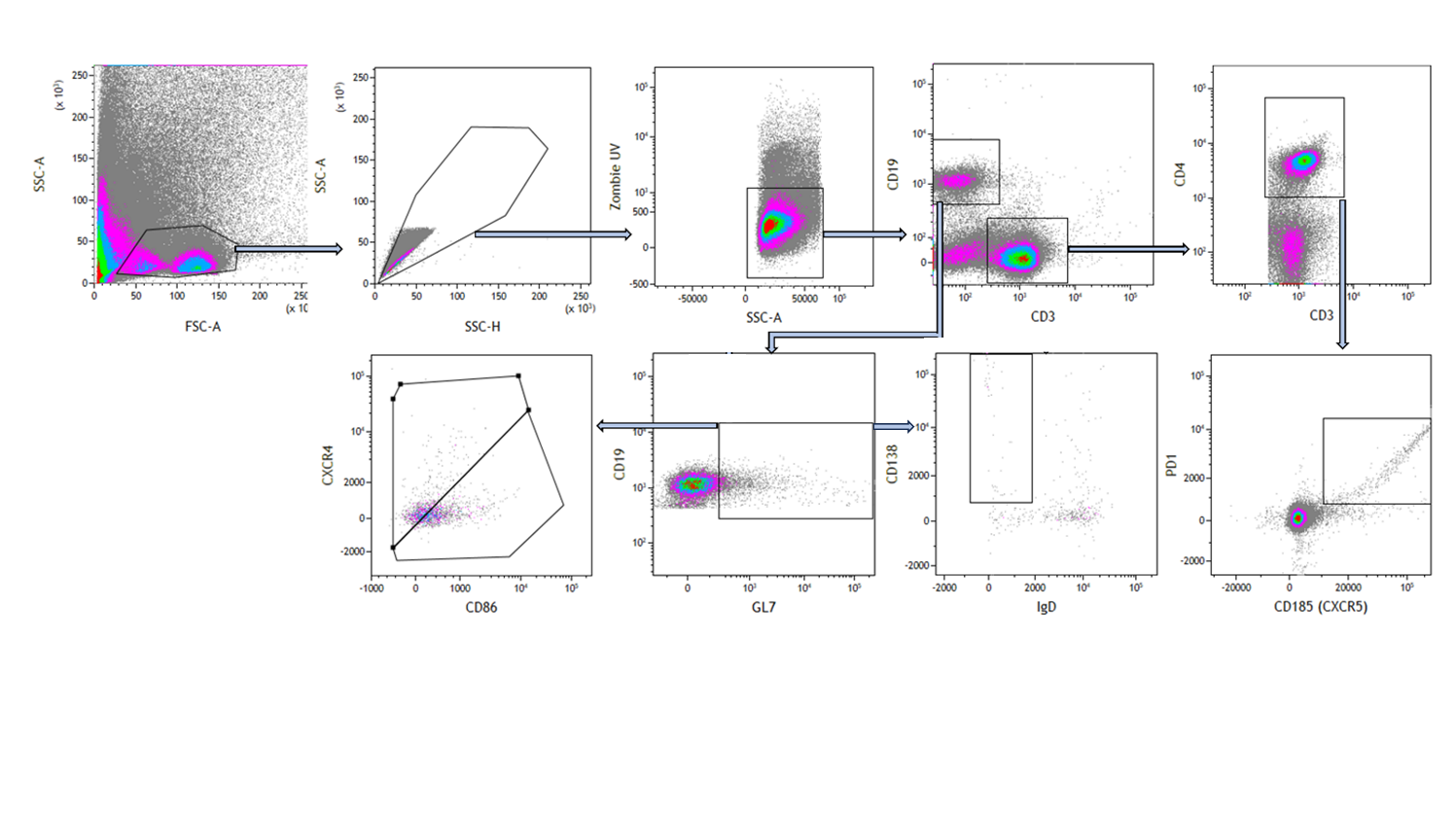


**Supplementary Figure 1.** Gating strategy.

**
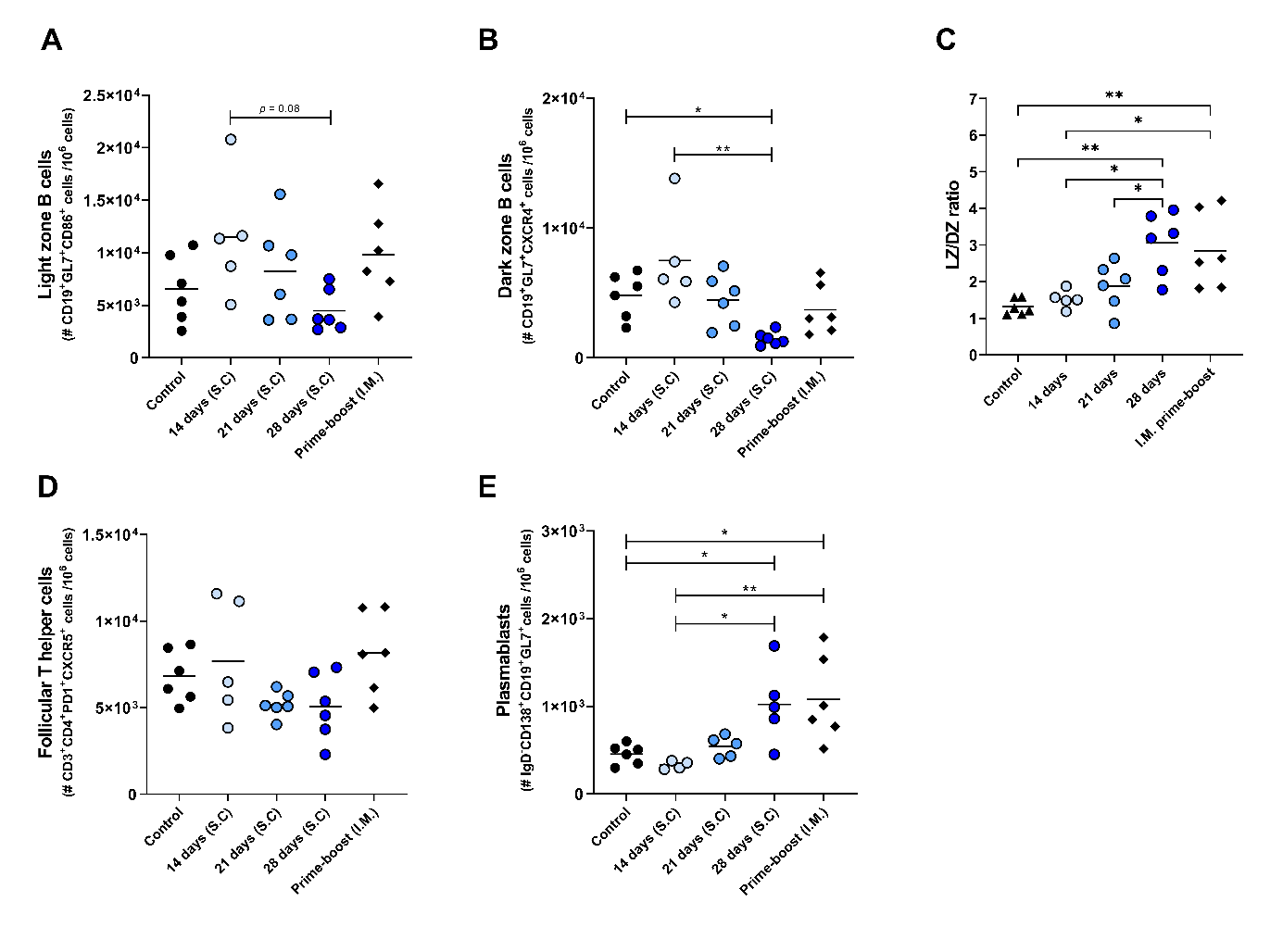
**

**Supplementary Figure 2.** Absolute numbers of immune cells in the germinal center. GC responses in lymph nodes of mice, treated as described in the legend of Figure 1, were assessed by measuring the absolute numbers of light zone B_GC_ cells (A), dark zone B_GC_ cells (B), Follicular T helper cells (D), and Plasmablasts (E). Panel C shows the LZ/DZ ratio calculated on basis of the cell numbers
